# A Pipeline for Predicting the Treatment Response of Neoadjuvant Chemoradiotherapy for Locally Advanced Rectal Cancer Using Single MRI Modality: Combining Deep Segmentation Network and Radiomics Analysis Based on “Suspicious Region”

**DOI:** 10.3389/fonc.2021.711747

**Published:** 2021-08-04

**Authors:** Xiaolin Pang, Fang Wang, Qianru Zhang, Yan Li, Ruiyan Huang, Xinke Yin, Xinjuan Fan

**Affiliations:** ^1^Department of Radiation Oncology, The Sixth Affiliated Hospital of Sun Yat-sen University, Guangzhou, China; ^2^Guangdong Institute of Gastroenterology, Guangdong Provincial Key Laboratory of Colorectal and Pelvic Floor Diseases, Supported by National Key Clinical Discipline, Guangzhou, China; ^3^School of Computer Science and Engineering, Sun Yat-sen University, Guangzhou, China; ^4^Department of Pathology, The Sixth Affiliated Hospital of Sun Yat-sen University, Guangzhou, China

**Keywords:** LARC, nCRT, MRI, radiomics analysis, deep learning

## Abstract

Patients with locally advanced rectal cancer (LARC) who achieve a pathologic complete response (pCR) after neoadjuvant chemoradiotherapy (nCRT) typically have a good prognosis. An early and accurate prediction of the treatment response, i.e., whether a patient achieves pCR, could significantly help doctors make tailored plans for LARC patients. This study proposes a pipeline of pCR prediction using a combination of deep learning and radiomics analysis. Taking into consideration missing pre-nCRT magnetic resonance imaging (MRI), as well as aiming to improve the efficiency for clinical application, the pipeline only included a post-nCRT T2-weighted (T2-w) MRI. Unlike other studies that attempted to carefully find the region of interest (ROI) using a pre-nCRT MRI as a reference, we placed the ROI on a “suspicious region”, which is a continuous area that has a high possibility to contain a tumor or fibrosis as assessed by radiologists. A deep segmentation network, termed the two-stage rectum-aware U-Net (tsraU-Net), is designed to segment the ROI to substitute for a time-consuming manual delineation. This is followed by a radiomics analysis model based on the ROI to extract the hidden information and predict the pCR status. The data from a total of 275 patients were collected from two hospitals and partitioned into four datasets: Seg-T (N = 88) for training the tsraUNet, Rad-T (N = 107) for building the radiomics model, In-V (N = 46) for internal validation, and Ex-V (N = 34) for external validation. The proposed method achieved an area under the curve (AUC) of 0.829 (95% confidence interval [CI]: 0.821, 0.837) on In-V and 0.815 (95% CI, 0.801, 0.830) on Ex-V. The performance of the method was considerable and stable in two validation sets, indicating that the well-designed pipeline has the potential to be used in real clinical procedures.

## Introduction

Colorectal cancer is currently still the third most common cancer and the second most fatal cancer in the world (1). Nearly 30% sufferers are rectal cancer patients (2), great numbers of which are in the locally advanced stage at initial diagnosis (3).

To date, for patients with locally advanced rectal cancer (LARC), neoadjuvant chemoradiotherapy (nCRT) followed by total mesorectal excision (TME) has been the standard clinical treatment (4–6). The purpose of nCRT is to improve the feasibility of surgical procedures for LARC and reduce the incidence of complications, as it not only improves the local tumor control rate but also exhibits less toxicity to the human body (7). Clinically, the pathological response of LARC patients after nCRT treatment has demonstrated obvious heterogeneity (8). For a large percentage of patients (approximately 70–80%), the tumor will have been found to be shrunken or down-staged, and some patients may even have complete regression. It has been reported that approximately 20% of patients, defined as pathologic complete response (pCR) patients, contain no residual surviving tumor cells after nCRT and surgery (9, 10). These patients have a favorable long-term prognosis with superb local control and disease-free survival (11). Therefore, for pCR patients, the option of organ-saving treatment could be developed to replace surgery. However, currently, the only way to accurately diagnose pCR is to utilize a pathological diagnosis after TME surgery, which presents an insoluble dilemma (12–14). As a result, a prediction method before surgery would greatly assist doctors in evaluating the treatment effects of nCRT and construct a tailored plan for each patient.

In recent years, magnetic resonance imaging (MRI) has been widely used as a non-genetic and non-invasive diagnostic method to assess the tumor condition due to its superior soft-tissue contrast and high spatial resolution. The T2-weighted (T2-w) MRI is recognized as the most important modality for rectal cancer assessments (15). Some previous works have attempted to evaluate the response of nCRT on T2-w MRI using assessments of post-treatment T staging (ymrT), tumor regression grading (mrTRG), volume reduction post-treatment, and other characteristics (16). However, the results were dependent on the experience of doctors, thus introducing subjectivity. The development of more objective ways to extract information from the MRI and guide clinical diagnosis is required.

Radiomics is a mathematical technique that utilizes high-throughput extraction of shape, intensity, and texture features from images, and transforms this visual information into high dimensional features for quantitative analysis (17). Radiomics analysis can help obtain additional image information with reliability and objectivity that may be invisible in human assessments (18). Applying a radiomics analysis to predict the pCR on an MRI has drawn increasing attention. In many studies (19–21), researchers have used a pre-nCRT MRI to analyze the relationship of the radiomics features and the pCR status. The use of a pre-nCRT MRI could provide a clear tumor region for analysis. However, considering that the nCRT could affect the tumor and change its properties, the use of a pre-nCRT MRI is indirect and may not reflect the true condition of the patient after nCRT treatment.

Analysis of a post-nCRT MRI might be a more direct method. However, the problem still remains that the region of interest (ROI) delineation on a post-nCRT is much more difficult due to tumor recession and the appearance of the fibrosis region. In previous studies (22, 23), researchers applied a pre-nCRT MRI to provide a reference of the primary tumor region or the treated region for a post-nCRT MRI. However, a pre-nCRT MRI is not always available in real clinical practice, as some patients may be diagnosed with LARC using proctoscopy, and some patients may be transferred from other hospitals without access to the two previously scanned MRIs. Currently, only a few of studies have utilized a post-nCRT MRI to predict the pCR status. The work of Horvat et al. (15) obtained considerable results by applying a radiomics analysis on a post-nCRT T2 and diffusion-weighted imaging (DWI) MRI; however, the ROI was still obtained due to a careful discussion by at least two experienced physicians. An accurate delineation might be difficult to obtain for less experienced physicians without the reference of a pre-nCRT MRI. Additionally, it is time-consuming and resource-wasting if each delineation requires at least two physicians in clinical practice. Inspired by this, the aim of this study is to explore a pipeline that only uses the information from a single post-nCRT T2 MRI combined with a new method to provide a fast and reliable ROI. Deep learning uses multiple layers as a portion of a broader family of machine learning methods and has been successfully applied to various medical tasks (24–28).

In this study, we introduce a deep learning model for ROI delineation. A novel two stage model, termed the two-stage rectum-aware U-Net (tsraU-Net), is proposed to replace human evaluation. The ROI should be feasible for a deep learning model to find and contain sufficient information relating to the pCR status; hence, it is defined on a continuous region having abnormal intensity signals on a T2-w MRI assessed by radiologists that has a high possibility to contain a tumor or fibrosis. It is considered a rougher region, as the further identification of a tumor, fibrosis, or other tissues like edema is not defined. The following analysis extracts a great number of radiomics features, including texture, first-order statistics, and shape, on the ROI and its wavelet decompositions to represent certain properties. Machine learning and statistical techniques are later applied to select the most representative features and construct a final model to predict the pCR status.

## Materials and Methods

### Patients

We included a total of 496 patients from multiple institutions who received nCRT treatment diagnosed with LARC. Patients were retrospectively enrolled from July 2011 to December 2018 from two hospitals (Guangdong Institute of Gastroenterology, Sixth Affiliated Hospital of Sun Yat-sen University and Yunnan Cancer Center). The inclusion criteria were as follows: (1) adenocarcinoma confirmed by pathologists (excluding mucinous adenocarcinoma); (2) tumor located within 15 cm from the edge of the anus; (3) received nCRT treatment and TME; (4) in the clinical stage of T3–4 or N-positive; (5) completed the restaging MRI; and (6) the restaging MRI was performed no more than 1 week before the TME. This clinical trial was approved by the Clinical Ethics Review Committee of Sixth Affiliated Hospital of Sun Yat-sen University (2020ZSLYEC-010).

**Figure 1:**
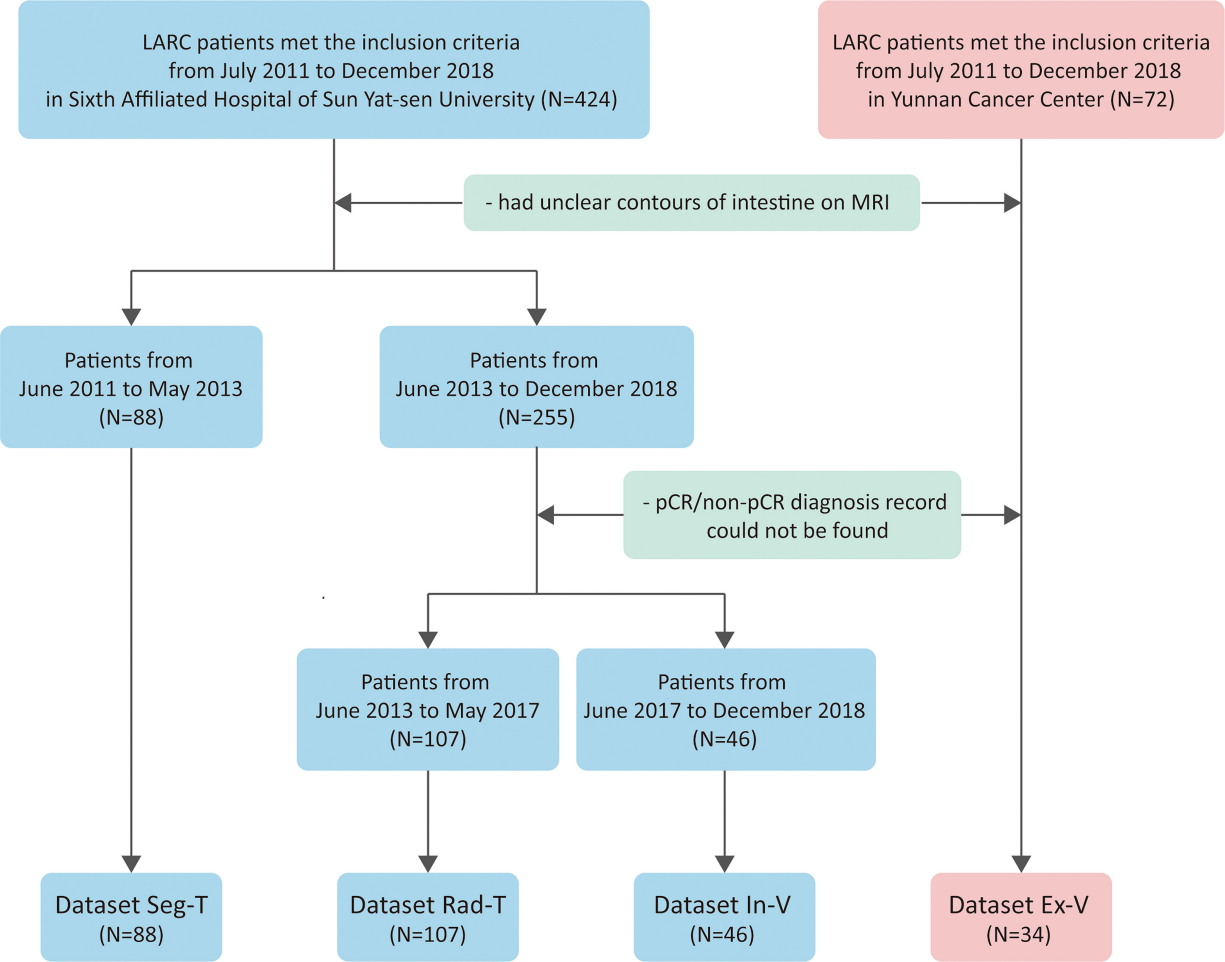
Flowchart showing how patients from two hospitals were collected and partitioned.

Patients from the Sixth Affiliated Hospital of Sun Yat-sen University were separated into three groups. Dataset Seg-T consisted of patients from July 2011 to June 2013 to train deep networks for ROI segmentation; dataset Rad-T consisted of patients from June 2013 to May 2017 to build the radiomics model for predicting the pCR status; and dataset In-V consisted of patients from June 2017 to December 2018 for internal validation. In addition, patients from Yunnan Cancer Center (dataset Ex-V) were used as an external validation set. Patients were further selected according to the following exclusion criteria: (1) poor MRI quality caused by severe inflammatory effusion, intestinal adhesions, or bowel movements, and (2) (for dataset Rad-T, Val, E-Val) the absence of a postoperative pathological diagnosis. **Figure 1** shows the flowchart of the patient selection process.

All of the patients’ treatments were discussed by the multidisciplinary team (MDT). Patients were delivered the intensity-modulated radiotherapy treatment (IMRT), and a dose of 45 Gy for 25 fractions was delivered to the clinical target volume. Then, a boost dose of 5.4 Gy was delivered to the gross tumor. The concurrent chemotherapy treatment was based on oral or intravenous 5-fluorouracil. Following the completion of neoadjuvant treatment, all of the patients received TME surgery. The majority of patients received adjuvant chemotherapy based on FOLFOX or CAPOX based on the decision of the members of the MDT.

### Pathology Assessment of Response

In this study, the pCR diagnosis was confirmed by two pathologists with more than 12 years of experience. Following the recommendation of the NCCN Guidelines for rectal cancer (29), patients with no surviving tumor cells in the surgical pathological specimens were judged as pCR; otherwise, they were judged as non-pCR.

### MRI Data Acquisition

All of the MRI images were scanned under a 1.5-Tesla MRI unit (30). Bowel preparation was not routinely used for most cases prior to the examination. However, some specific patients with relatively small tumors in the sagittal view were filled with some rectal gel, making it easier to identify tumors on the oblique axis. The MRIs in the datasets Seg-T, Rad-T, and In-V (Sixth Affiliated Hospital of Sun Yat-sen University) were acquired using GE OPTIMA MR360 with a 100 ms echo time, a 4000 ms repetition time, a 100 field of view, a 512 × 512 matrix, 0.4–0.5 pixel spacing, and 5 mm slice thickness. The MRIs in the dataset Ex-V (Yunnan Cancer Hospital) were acquired using Philips Ingenia with a 100 ms echo time, a 4000 ms repetition time, a 100 field of view, a 432 × 432 matrix, 0.4–0.5 pixel spacing, and 5 mm slice thickness.

### Data Pre-Processing

As suggested by some researchers (31), we applied complex methods to the pre-process MRI to both improve the image quality and unify the geometric and intensity patterns with the aim to assure the success of our analysis. The steps included (1) all of the MRIs were resampled into 0.4 mm × 0.4 mm pixel spacing using bilinear interpolation; (2) the size of each image matrix was unified into 544 × 544 by cutting or padding the background; (3) the intensity of each patient was adjusted using BiasCorrection to remove any inhomogeneity; and (4) the intensity histogram of each patient was matched to one selected patient (as template) who was from Seg-T. All of the procedures were implemented using the open-source python package “SimpleITK” (32).

### Suspicious Region Definition

**Figure 2:**
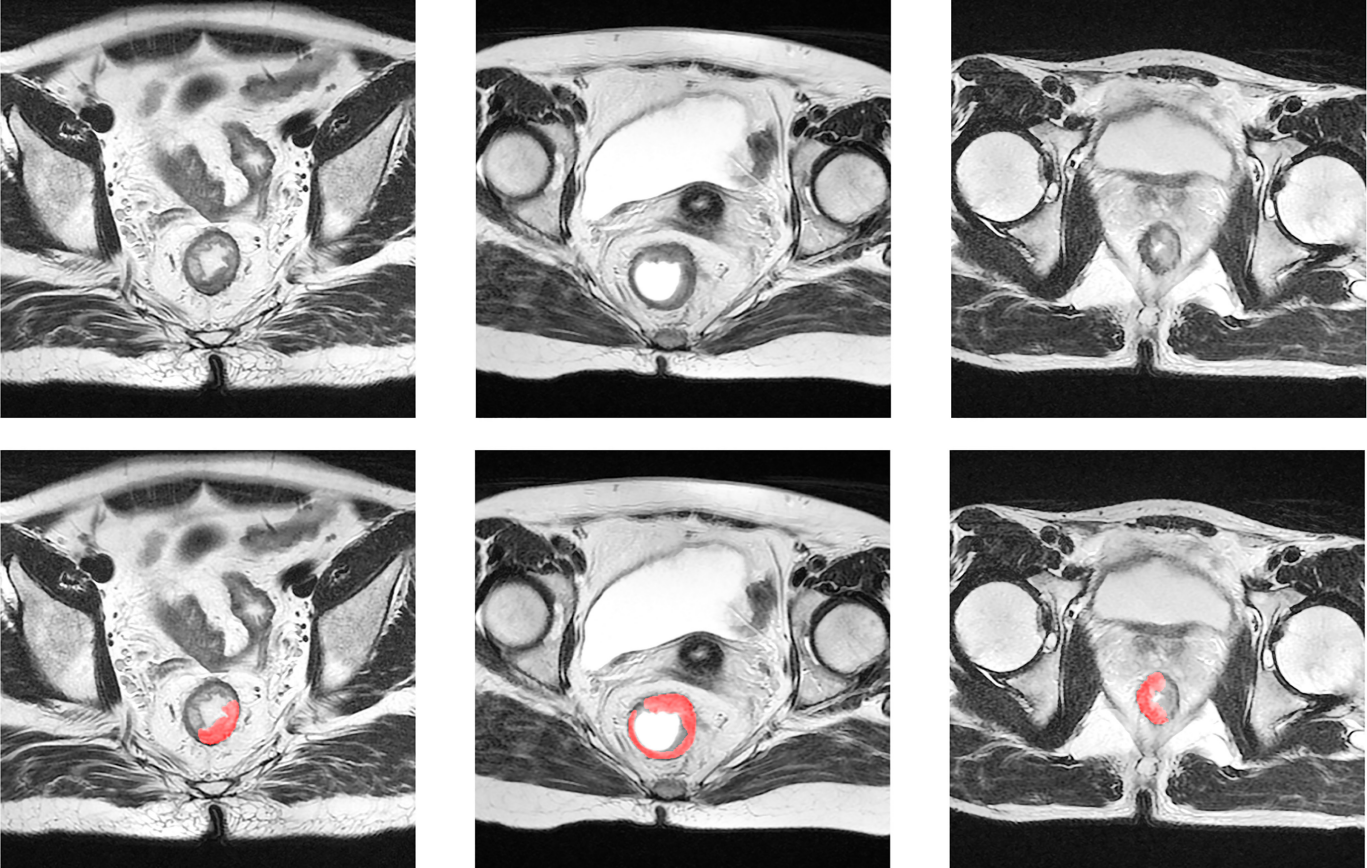
Examples of suspicious region. The first row is the original T2-w MRI, and the second row displays the delineation of the suspicious region in red color.

The “suspicious region” in our study was defined as a continuous region containing 129 abnormal intensity signals compared to a normal rectal wall, which are highly suspected to be cancer or fibrosis according to clinical experience. Following the guidance (33), the abnormal signals may have presented as slightly high, low, or mixed intensities. By such definition, the exact cancer and fibrosis region was not further distinguished. Instead, we relied on the radiomics analysis to elicit the hidden properties of the cancer or fibrosis and predict the pCR. In particular, the “suspicious region” was delineated by radiologists on only a post-nCRT T2-w MRI. As the region was visible to human vision, we assumed it could be captured by deep learning as well. Therefore, the use of “suspicious region” was both sufficient and proper in the pipeline that combined a radiomics analysis and deep learning. **Figure 2** provides some examples of the suspicious region.

### Deep Learning-Based Segmentation

#### The tsraU-Net Model

**Figure 3:**
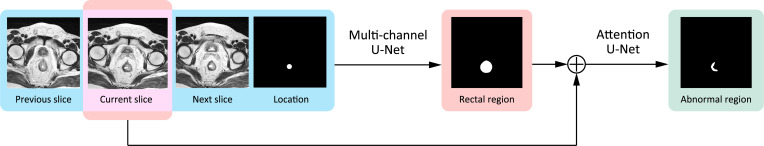
The framework of tsraU-Net model.

To provide a reliable ROI using deep learning for the radiomics analysis, the most important consideration was rectum localization. If the deep segmentation network misrecognized the rectum with other organs such as the colon, uterus, bladder, or prostate due to a morphology change or location shift of the rectum, it could still find a “suspicious region,” but not related to the pCR at all, thereby making the radiomics analysis totally meaningless. To address such a problem, we proposed a two-stage model, named the two-stage rectum-aware U-Net (tsraU-Net), which would first find the rectum region and then segment the ROI using the awareness of the rectum location. The overall framework of the tsraU-Net model is shown in **Figure 3**.

U-Net, a deep segmentation network expressly designed for biomedical segmentation tasks (24), was applied as a base model in both of the two stages of the tsraU-Net. Further improvements were made in each stage according to the task. The detailed descriptions were organized as follows. First, we briefly introduced the base model, U-Net. Next, we provided comprehensive explanations of the improvements in the two stages of tsraU-Net. Finally, we described other adjustments of the base model.

##### The Original U-Net Model

The original U-Net is a fully convolution network (FCN) containing an encoder to extract features and a decoder to reassemble features. Typically, both of them have five convolution blocks, s.t. each block consists of two 3 × 3 convolutions and a rectified linear unit (ReLU) for activation. After going through the convolution block, the number of features (more specifically, channels) would double in the encoder part and halve in the decoder part symmetrically. Between each convolution block in the encoder, the max pooling operation is applied to reduce the image resolution. Oppositely, an up-sample operation is inserted into the neighboring convolution blocks in the decoder to increase the image resolution. To fully utilize high resolution information, high resolution features in the encoder are concatenated to the corresponding convolution blocks in the decoder. This is named a “skip connection.” In addition, a 3 × 3 convolution is applied after the last convolution block in the decoder to combine the rest of the features and obtain the final segmentation result.

##### The First Stage

In the first stage, we designed a four-channel 2D U-Net aiming to guide the segmentation with a plentiful amount of information. Knowing that rectal regions would maintain certain continuity between neighboring MRI slices, the input of our model contains not only the currently input MRI slice but also its previous slice and the next slice to help detect the contours of the rectum. If the rectal wall is unclear compared with the neighboring region on the current slice, the other two slices may provide extra information. Furthermore, for each patient, we roughly marked two to four points inside the rectum for localization. Initially, the first and last MRI slices were given localization points, with bilinear interpolation applied between them to give localization information to the middle layers. If the shape of the rectum was not regular, another one to two points will be given in the middle layers. The previous slice, the current slice, the next slice, and the position information were combined into four channels as the input of our first stage model. The output of the first stage was the region of the rectum.

##### The Second Stage

**Figure 4:**
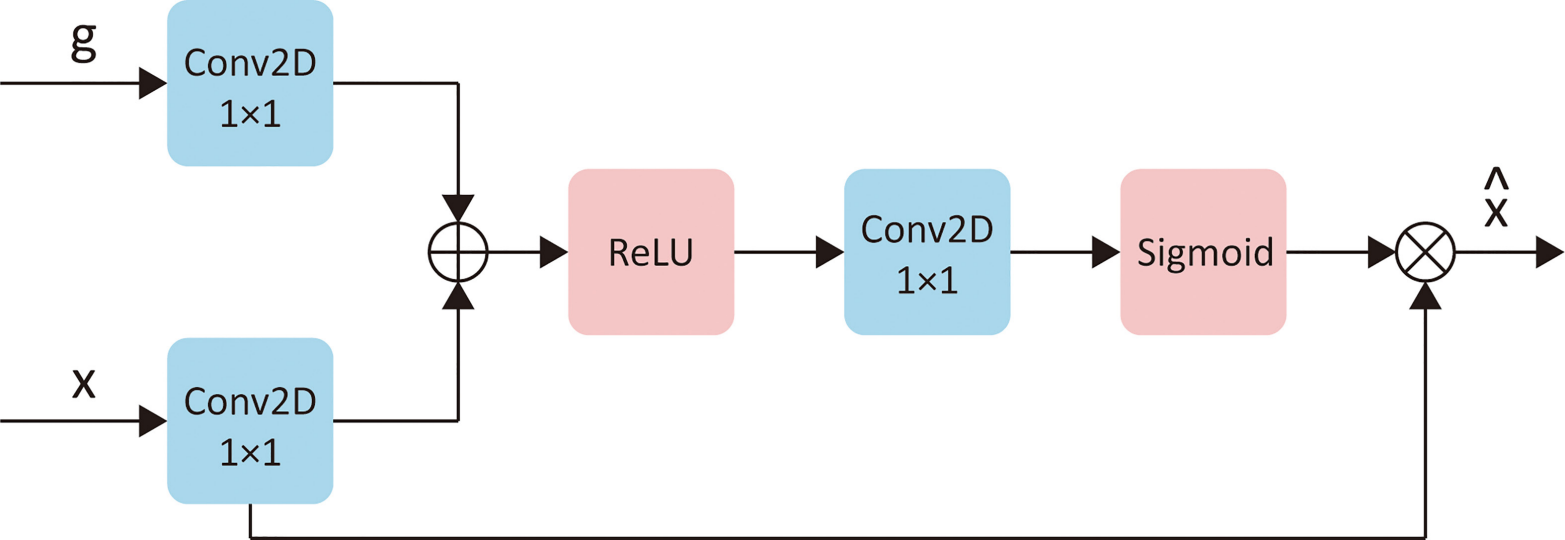
The detailed explanation of attention gates. Let x represents the “skip connect” features from encoder, g represents the features from corresponding former decoder layer, then $\hat{x}$ is the updated of x. “Conv2D 1×1” represents a 2D convention with kernel size of 1×1. “ReLU” and “Sigmoid” are two different activation functions.

The second stage would use the currently inputted slice and the predicted rectal region in the first stage to find the “suspicious region.” In this stage, we focused on strengthening the model awareness of the abnormal intensity signals and, hence, applied an “attention” mechanism. “Attention” was first introduced in the natural language process (NLP) tasks to encourage models to pay more attention to efficacious information and suppress irrelevant information. There are two types of attention, i.e., soft attention and hard attention. In this task, we used the soft attention mechanism for the model (34). This method would update the propagated features from “skip connection” by point-wise multiplication with a weight matrix given by an attention gate. An attention gate is a block containing several 1 × 1 convolutions and activation functions. It uses both the propagated features and the features from the corresponding former decoder block as input. **Figure 4** provides a detailed explanation of an attention gate. Specifically, we inserted four attention gates into the original U-Net model s.t. every “skip connection” was followed by an attention gate.

##### Adjustments on Base Model

In addition to the above methods, we also made some small adjustments to the original U-Net model: (1) the addition of image padding during convolution so that the image size would not change; (2) the addition of instance normalization after each convolution block to accelerate the convergence; and (3) the replacement of the ReLU activation function with the Leaky Rectified Linear Unit (Leaky ReLU) to prevent the vanishing gradient problem.

#### Loss Function

The loss function in both two stages is the Dice Loss, which is widely used in medical image segmentation. It is defined from the dice coefficient, which essentially measures the overlap of two sets. The dice coefficient has a range of 0–1, where 1 means complete overlap. It is defined as Equation 1:

(1) $$\text{Dice}=\frac{2\left|A\cap B\right|}{\left|A\right|+\left|B\right|}$$

where *A* ∩ *B* is the intersection of sets A and B, | | represents the number of elements in the set.

As for Dice Loss, it is simply defined by the following Equation 2.

(2) Dice Loss=1−Dice

#### Experiment Setup

To provide the gold standard of segmentation, two radiologists (one had 6 years of experience and one had 9 years of experience) reviewed the post-nCRT T2-w MRI of the Dataset Seg-T and InV (for validating the performance), and they jointly provide delineations of the rectum and the “suspicious region.” When delineating the “suspicious region,” the normal rectal wall should be avoided, and for some patients with rectal gel filling, the gel should also be avoided. Both radiologists were completely blinded to the histopathology information, as well as the pre-nCRT MRI of patients. Following the instructions in the guidance (33), they used the rectal wall as a reference to find abnormal signals. This work was performed *via* ITK-snap version 3.4.0 software (http://itk-snap.org).

While training, the initial hyper-parameters were established identically in two stages. The optimizer we used was the Adaptive Moment Estimation (Adam) (35) with an initial learning rate *α* = 2 × 10^-4^, *β* = (0.9,0.999) and would decay 30% every 20 epochs. The maximum training epoch was set to 120, the early stopping method was applied to prevent overfitting, and the training would stop if performance on the minority set did not improve over 30 epochs. The model was implemented with PyTorch 1.8.1 (36) on a Nvidia Titan X GPU with 12 G of memory.

### Radiomics Analysis

After the segmentation model was well-trained, it was directly applied to the Rad-T, In-V, and Ex-V to obtain the ROI. Then, the following procedures of the radiomics analysis were applied to build the pCR prediction model.

#### Feature Extraction

In order to extract useful information related to the pCR status, a large feature space was generated, which included features not only from the original image but also from its wavelet decomposition images. A total of 93 types of features were calculated on each original image and its four Harr wavelet subbands, i.e., HH, HL, LH, and LL. The 93 features include 18 intensity features and 75 texture features. In addition, nine shape features were extracted on the original image. Together, 474 features were generated from each MRI slice.

Concerning that each patient had a different number of MRI slices, we used the arithmetic mean, three quartiles (Q1, Q2, and Q3 points), and the standard deviation of the features extracted from all of the slices as representations. Therefore, each patient had 2370 features in total.

All of the features were extracted using the Python package “PyRadiomics” (37). As announced in its document, most features meet the Introduction Intellidyne Business Systems (IBSI) standard, which would increase the reliability to our experiments. More details are shown on the “PyRadiomics documentation” website (http://pyradiomics.readthedocs.io).

#### Feature Selection

Two feature selection steps were applied in our study to increase the robustness and avoid overfitting. First, we evaluated the discriminative power of each single feature by calculating the Harrell’s concordance index (C-index) (38) between the features and the pCR status. The univariate Cox analysis is a commonly used procedure in survival analysis, and we adopted this method because it does not require the features to follow a normal distribution compared with a *t*-test. Let *A* = {*α*_1_, *α*_2_, …, *α_n_*} denote the features of patients, *β* present the pCR status of patients, for each feature *α_f_* ∈ *A*, the C-index can be computed with the Equation 3.

(3) $$C-\text{index}(\alpha_{f},\beta )=\frac{\Sigma_{i,j}U(\alpha_{fj}<\alpha_{fi})\cdot U(\beta_{j}<\beta_{i})}{\Sigma_{i,j}U(\beta_{j}<\beta_{i})},$$

where *α_fi_* is the *i*-th value of *α_f_*, *β_i_* is the *i*-th value of *β*, and *U* (*a* < *b*) = 1 if *a* < *b* else 0.

By definition, C-index equals to 1 means the best discriminative power and C-index equals to 0.5 represents a theoretically result of random prediction. We calculate the maximum of C-index(*α_f_*, *β*) and C-index(–*α_f_*, *β*) as the predictive score of feature *α_f_*. After calculation, scores are sorted and features with lower score are excluded.

In the second step, the remaining features were put into the least absolute shrinkage and selection operator (LASSO) (39) for further selection. LASSO is a logistic regression model with L1 regularization as a penalty of the coefficients. It will encourage the regression use of sparse features. The objective function of LASSO is:

(4) $$\min\frac{1}{2}{\left||Ax-\beta |\right|}_{2}^{2}+\lambda {\left||x|\right|}_{1},$$

where *A* is the matrix of radiomics features, *x* is the coefficient of each feature, *β* is the pCR status, and *λ* is the regularization penalty coefficient.

Due to the L1 regularization, the LASSO forces the sum of the absolute value of the regression coefficients to be less than a fixed value, minimizing the residual sum of the squares. Such an operation forces the certain coefficient to zero. After the LASSO regression, features with a coefficient of non-zero are retained. Here, *λ* was determined using a grid search and 5-fold cross validation on 100 iterations between 0.01 and 0.2.

#### The pCR Status Prediction

In our study, the support vector machine (SVM) (40) was applied to predict the pCR status. As suggested in study (41), the radial basis function (RBF) kernel was used. The RBF kernel is defined as Equation 5:

(5) $$K(a,b)=e^{\left(-\gamma {\left||a-b|\right|}^{2}\right)}$$

where *a*, *b* are two samples from dataset and γ is a hyper-parameter. γ and the regularization coefficient *C* of SVM were determined also by grid search on 5-fold cross validation within set {1/16, 1/8, 1/4, 1/2, 1}. After choosing the best γ and *C*, the SVM model was trained for the pCR prediction.

### Performance Evaluation

Various evaluation metrics of suspicious region segmentation and pCR prediction are listed below.

Three metrics—dice coefficient, sensitivity, and specificity—were applied to evaluate the performance of segmentation. The dice coefficient’s definition has been given in Equation 1. Sensitivity (SEN) and specificity (SPC) are defined as Equation 6 and Equation 7, where TP, FP, TN, and FN denoted true positive, false positive, true negative, and false negative, respectively.

(6) $$\text{SEN}=\frac{TP}{TP+FN}$$

(7) $$\text{SPC}=\frac{TN}{TN+FP}$$

As for the pCR status prediction, five metrics—the area under receiver operating characteristic (ROC) curve (AUC), accuracy, sensitivity, specificity, and the F-score—were applied for the evaluation. The F-score is a weighted harmonic mean that comprehensively considers sensitivity and specificity, which can be calculated as Equation 8.

(8) $$F_{\beta }-score=(1+\beta^{2})\cdot \frac{SEN\times SPC}{\beta^{2}\cdot SPC+SEN}$$

Here, we included F_0.5_, F_1_, and F_1.5_ in order to provide a multiple trade-off between specificity and sensitivity under different situations.

## Results

### Clinical Characteristics

In our study, 241 of 424 patients from Guangdong Institute of Gastroenterology, Sixth Affiliated Hospital of Sun Yat-sen University and 34 of 72 patients from Yunnan Cancer Center met the inclusion criteria and did not meet the exclusion criteria. After selection, the number of patients in SegT, Rad-T, In-V, and Ex-V were 88, 107, 46, and 34, respectively. More clinical information of the Rad-T, In-V, and Ex-V groups is provided in **Table 1**. Statistical comparisons were performed for each clinical characteristic between the two response groups (pCR vs. non-pCR). There were no statistical differences between the pCR and non-pCR in sex, age, and the pre-CRT N stage in all three sets.

**Table 1:** The clinical characteristics of patients in dataset Rad-T, In-V, and Ex-V.

Characteristics	Dataset Rad-T		*P-value*	Dataset In-V		*p-value*	Dataset Ex-V		*p-value*
	pCR^a^ (n = 36)	NonpCR (n = 71)		pCR (n = 8)	NonpCR (n = 38)		pCR (n = 6)	NonpCR (n = 28)	
Sex			0.72			0.24			0.18
Male	8 (22.2%)	18 (25.4%)		5 (62.5%)	31 (81.6%)		2 (33.3%)	19 (67.9%)	
Female	28 (77.8%)	53 (74.6%)		3 (37.5%)	7 (18.4%)		4 (66.7%)	9 (32.1%)	
Age (mean ± SD^b^, year)	53.4 ± 12.1	56.9 ± 9.9	0.11	57.9 ± 8.4	55.0 ± 11.1	0.49	60.0 ± 10.8	59.2 ± 8.2	0.83
Pre-CRT^c^ T stage			0.51			<0.01			0.31
T0	0 (0%)	0 (0%)		0 (0%)	0 (0%)		0 (0%)	0 (0%)	
T1	0 (0%)	0 (0%)		0 (0%)	0 (0%)		0 (0%)	0 (0%)	
T2	1 (2.8%)	3 (4.2%)		0 (0%)	0 (0%)		1 (16.7%)	0 (0%)	
T3	31 (86.1%)	55 (77.5%)		8 (100%)	30 (78.9%)		2 (33.3%)	8 (28.6%)	
T4	4 (11.1%)	13 (18.3%)		0 (0%)	8 (21.1%)		3 (50.0%)	20 (71.4%)	
Pre-CRT N stage			0.59			0.2			0.33
N0	4 (11.1%)	13 (18.4%)		0 (0%)	13 (34.2%)		1 (16.7%)	1 (3.6%)	
N1	17 (47.2%)	29 (40.8%)		4 (50.0%)	9 (23.7%)		1 (16.7%)	1 (3.6%)	
N2	15 (41.7%)	29 (40.8%)		4 (50.0%)	16 (42.1%)		4 (66.6%)	26 (92.8%)	
Post-CRT T stage			< 0.01			<0.01			< 0.01
T0	36 (100%)	0 (0%)		8 (100%)	0 (0%)		6 (100%)	0 (0%)	
T1	0 (0%)	7 (9.9%)		0 (0%)	2 (5.3%)		0 (0%)	4 (14.4%)	
T2	0 (0%)	18 (25.4%)		0 (0%)	11 (28.9%)		0 (0%)	6 (21.4%)	
T3	0 (0%)	43 (60.6)		0 (0%)	22 (57.9%)		0 (0%)	9 (32.1%)	
T4	0 (0%)	3 (4.2%)		0 (0%)	3 (7.9%)		0 (0%)	9 (32.1%)	
Post-CRT N stage			0.01			<0.01			0.17
N0	36 (100%)	50 (70.4%)		8 (100%)	30 (78.9%)		6 (100%)	21 (75.0%)	
N1	0 (0%)	20 (28.2%)		0 (0%)	8 (21.1%)		0 (0%)	6 (21.4%)	
N2	0 (0%)	1 (1.4%)		0 (0%)	0 (0%)		0 (0%)	1 (3.6%)	
Post-CRT CRM^d^			0.48			/			/
Negative	36 (100%)	70 (98.6%)		8 (100%)	38 (100%)		6 (100%)	28 (100%)	
Positive	0 (0%)	1 (1.4%)		0 (0%)	0 (0%)		0 (0%)	0 (0%)	

a pCR, pathologic complete response. ^a^SD, standard deviation. ^c^CRT, chemoradiotherapy, ^d^CRM, circumferential resection margin.

### Segmentation Performance

When training the segmentation model, Seg-T was randomly separated into two sets with percentages of 70% and 30%. The s.t. of the majority set was used for updating the model, and the minority set was used for selecting the best network parameters. After training, the model was validated on In-V, and the results are listed below.

#### The First Stage

The numerical results of stage one, segmentation of the rectum, are presented in **Table 2**. The results of the original U-Net are also provided for comparison. It can be seen that the 4-channel U-Net had a significant improvement compared to the original U-Net on the rectum segmentation. Moreover, as the 4-channel U-Net achieved (0.942, 0.965) 95% CI of the dice coefficient, it could be inferred that this network could provide a stable 300 and accurate rectum segmentation.

**Table 2:** Comparison between U-Net and 4-channael U-Net in the dice coefficient, sensitivity and specificity between the gold standard and the results from stage one, that is, 4-channel U-Net, in tsraU-Net as well as the baseline, original U-Net.

Model	Dice	Sensitivity	Specificity
U-Net	0.861	0.876	0.867
	(95% CI^a^: 0.850, 0.873)	(95% CI: 0.864, 0.888)	(95% CI: 0.852, 0.882)
4-channel	0.954	0.967	0.96
U-Net	(95% CI: 0.942, 0.965)	(95% CI: 0.955, 0.980)	(95% CI: 0.945, 0.976)

^a^CI, confidence intervals.

**Figure 5:**
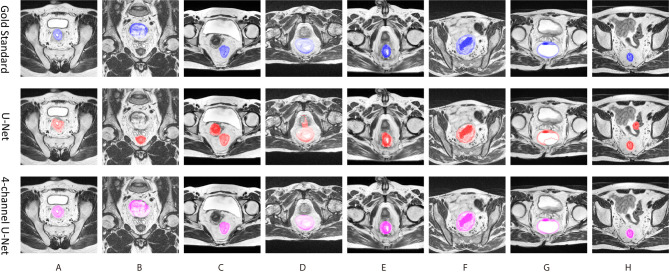
Eight typical cases of ructum segmentation **(A–H)**. The first row provide the gold standard, the second is the prediction of original U-Net, and the third row is the results of the first stage of tsraU-Net.

The visual results of the 4-channel U-Net and the original U-Net are also presented. **Figure 5** shows eight typical cases of rectum segmentation. The 4-channel U-Net had successfully segmented the rectum regions in all of the cases, while the U-Net showed different defects. In case (A), the U-Net was able to find the rectum, but the morphology lacked accuracy. In case (B), because the U-Net had no position information, the prostate was mistakenly judged as the rectum. In case (C), the U-Net outputted a continuous region containing both the rectum and the uterus. In case (D), due to the unclear rectal wall, the U-Net produced a bad result with an undesirable shape. In case (E), the prediction of the U-Net was less regular compared with the 4-channel U-Net. In case (G), the U-Net seriously under-segmented the rectum region. In case (H), the U-Net found two separated regions with similar sizes. Among those defects, (A) and (E) might be improved by post-processing methods, but for the rest, even postprocessing methods such as image dilation or the removal of the smaller region seems useless to obtain the correct rectum region. Thus, our design in the first stage has great importance for guaranteeing the success of the following analysis.

#### The Second Stage

Numerical results of stage two, segmentation of the “suspicious region,” are presented in **Table 3**. For a fair comparison, the U-Net model in this stage was also given the rectum localization information from the first stage, and we intended to evaluate the use of the attention mechanism. From the results, the attention slightly improved the result. The dice coefficient and specificity may not be considerably high, but the sensitivity achieved nearly 0.8, indicating that the network could find the major portion of the “suspicious region.”

**Table 3:** Comparison between U-Net and Attention U-Net in dice, sensitivity and specificity.

Model	Dice	Sensitivity	Specificity
U-Net	0.656	0.781	0.624
	(95% CI^a^: 0.630, 0.683)	(95% CI: 0.750, 0.812)	(95% CI: 0.590, 0.659)
4-channel	0.66	0.785	0.632
U-Net	(95% CI: 0.628, 0.691)	(95% CI: 0.752, 0.817)	(95% CI: 0.594, 0.668)

^a^CI, confidence intervals.

**Figure 6:**
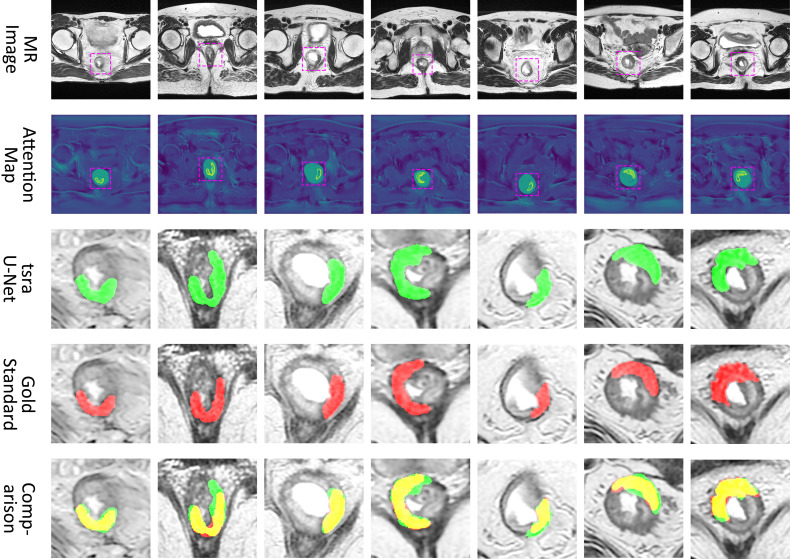
Attention map and comparison of suspicious region segmentation between gold standard and tsraU-Net. The first row is the input of the network, the second row is the attention map provided by attention U-Net, the third row is the prediction of tsraU-Net, the fourth row is the gold standard, and the last row is the overlapped region between prediction and gold standard.

**Figure 6** shows a visual display of the tsraU-Net, including the last attention maps, the final segmentation results, and the overlapping region compared with the gold standard. In some cases, such as (B), (D), and (F), the segmentation results are oversized. However, the morphology between them is still similar, and the segmentation results do not neglect most of the gold standard. Consequently, we believe the model is capable of providing the ROI with enough information for radiomics analysis.

### Treatment Response Prediction

**Figure 7:**
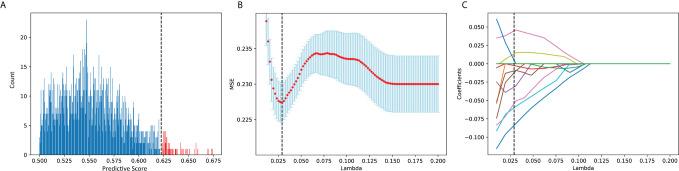
**(A)** Histogram of predicted score. Features in red were remained and in blue were excluded. **(B)** MSE of each *λ* in LASSO while grid search. **(C)** The coefficient of each feature in lasso while grid search.

A total of 2370 features representing certain properties of the “suspicious region” predicted by tsraUNet were extracted from each patient. These features were progressively selected in the initial univariate analysis, and only approximately the top 2.5% features, which was 63 features, remained according to their predictive scores. The histogram of the predicted scores with the number of features is illustrated in **Figure 7A**. A distinct gap was found between the remaining features and the excluded features with a corresponding threshold of 0.622.

The remaining features were then put into LASSO. After a grid search and cross-validation, the best *λ* was 0.293. The grid search of *λ* in the LASSO regression to minimize the residual mean square error (MSE) is visually provided in **Figures 7B, C**, which provides the coefficients of the features during the grid search. Ten features were finally chosen, and they are presented in **Table 4**. The detailed descriptions of these features can be found in the “PyRadiomics documentation.”

**Table 4:** The features finally remained, and their coefficients.

Features Group	Abbreviation	Attribute	Coefficient
original firstorder	Maximum	Q2	-0.03166
original firstorder	Maximum	Q3	-0.009714
original glcm	MCC	SD	-0.083918
original gldm	LargeDependenceHighGrayLevelEmphasis	Q2	-0.002476
original gldm	LowGrayLevelEmphasis	Q1	0.044713
wavelet-LH ngtdm	Busyness	Q1	-0.012269
wavelet-LH ngtdm	Busyness	Q2	-0.054026
wavelet-LH ngtdm	Strength	Q2	0.013605
wavelet-HL firstorder	Median	AVG	-0.059893
wavelet-LL glszm	LargeAreaLowGrayLevelEmphasis	Q3	0.004648

**Figure 8:**
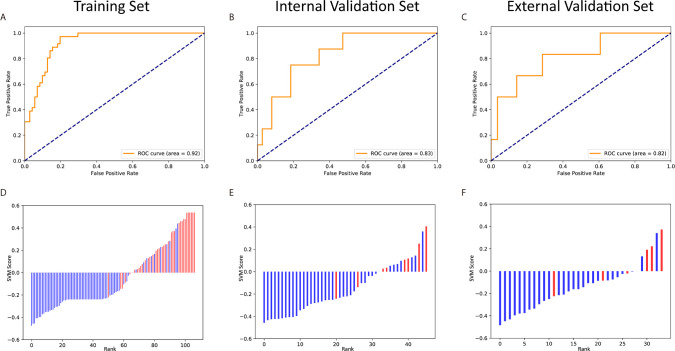
The ROC curves and scores of predicting pCR in the training and validation sets. The ROC curves of the training set **(A)**, internal validation set **(B)** and external validation set **(C)**. The scores of the training set **(D)**, internal validation set **(E)** and external validation set** (F)**.

The remaining 10 features was used to build a SVM classifier. The hyperparameters were decided after grid search and cross-validation: *C* = 1 and γ = 0.125. After training, the SVM achieved 0.924 of the AUC on Rad-T, 0.829 on In-V, and 0.815 on Ex-V. More numerical results are displayed in **Table 5**. In addition, **Figure 8** gives a visual display of the AUC and SVM scores [provided by the Python package “scikit-learn” (42)].

**Table 5:** The pCR^a^ status predicted performance on datasets Rad-T, In-V and Ex-V, in terms of AUC, accuracy, sensitivity, specificity, F_0.5_-score, F_1_-score, F_1.5_-score.

Dataset	AUC^b^	Accuracy	Sensitivity	Specificity	F0.5-score	F_1_-score	F_1.5_-score
Rad-T	0.924	0.860	0.861	0.859	0.860	0.860	0.860
	(95% CI^c^:	(95% CI:	(95% CI:	(95% CI:	(95% CI:	(95% CI:	(95% CI:
	0.923, 0.926)	0.856, 0.863)	0.855, 0.867)	0.855, 0.863)	0.856, 0.863)	0.820, 0.880)	0.825, 0.901)
In-V	0.829	0.804	0.750	0.816	0.802	0.782	0.769
	(95% CI:	(95% CI:	(95% CI:	(95% CI:	(95% CI:	(95% CI:	(95% CI:
	0.821, 0.837)	0.794, 0.815)	0.720, 0.780)	0.805, 0.827)	0.789, 0.811)	0.689, 0.793)	0.722, 0.794)
Ex-V	0.815	0.853	0.500	0.929	0.793	0.650	0.583
	(95% CI:	(95% CI:	(95% CI:	(95% CI:	(95% CI:	(95% CI:	(95% CI:
	0.801, 0.830)	0.841, 0.865)	0.453, 0.548)	0.919, 0.938)	0.746, 0.813)	0.634, 0.678)	0.555, 0.615)

a pCR, pathologic complete response. ^b^AUC, area under the curve; ^c^CI, confidence intervals.

We further applied *t*-test on the three datasets to test the distribution of SVM score between real pCR and non-pCR patients. The *p*-values on Rad-T, In-V, and Ex-V are 1.26×10^–10^, 1.41×10^–3^, and 3.26×10^–3^, respectively, indicating that the SVM score between real pCR and non-pCR is from different distribution under significance level *α* = 0.05.

**Figure 9:**
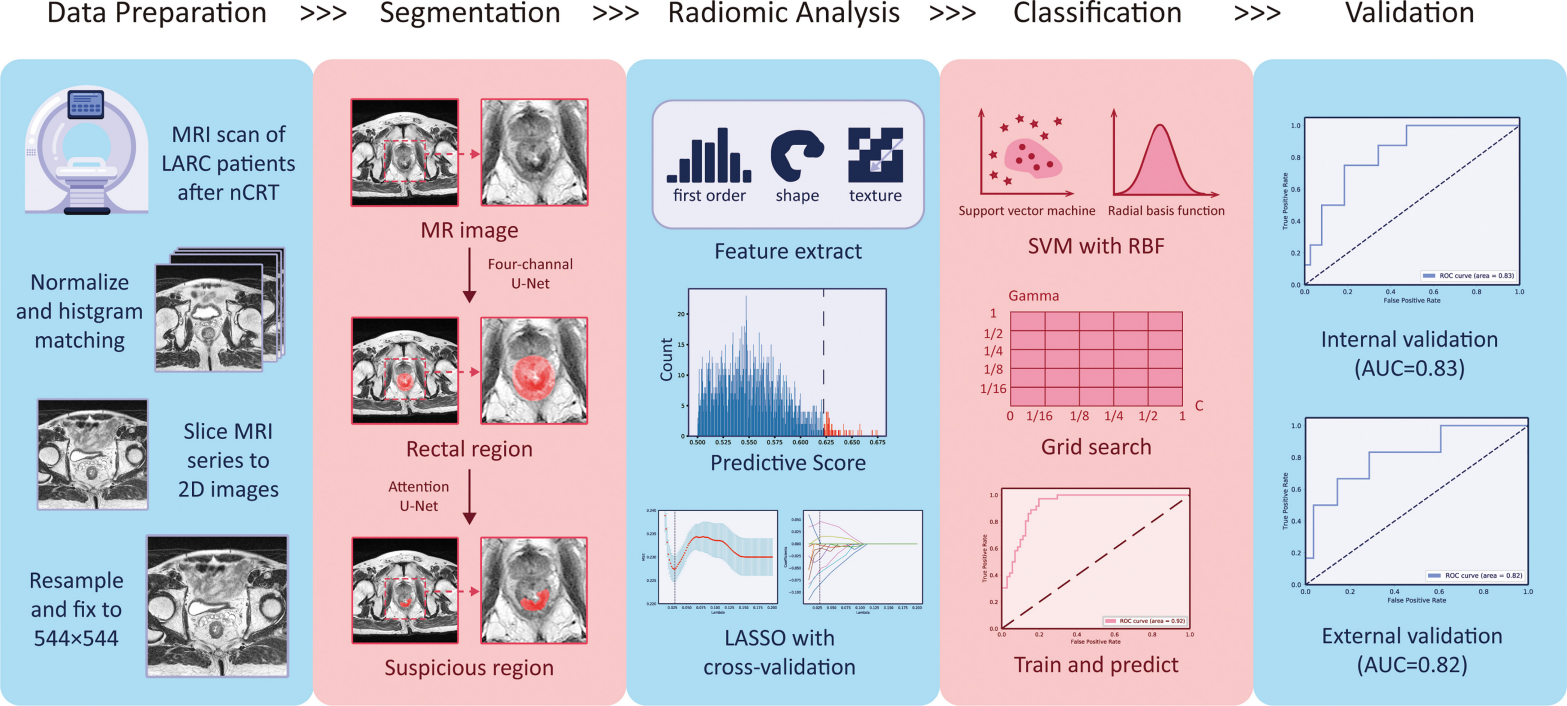
The overall workflow in this study.

From the above results, we conclude that the radiomics model could extract information related to the pCR and predict its status with certain reliability. Furthermore, the results indicated that the “suspicious region” is capable to be the ROI in this research, which means a single post-nCRT T2-w MRI has the ability to predict the pCR status without the help of a pre-nCRT MRI or other post-nCRT modalities. The overall pipeline is provided in **Figure 9**.

## Discussion

In this study, we proposed a method for predicting the pCR status of LARC patients after nCRT that only requires the post-nCRT T2-w MRI and a few manual operations. We provided a novel definition of the suspicious region and used it as the ROI to build the radiomics analysis. Furthermore, we designed a deep learning model for suspicious region segmentation that could greatly reduce the workload of radiologists. Our experimental results, 0.829/0.815 AUC on the internal/external validation set, prove the feasibility and stability of our method in pCR prediction, indicating that our method has great potential for providing assistance to doctors in clinical diagnosis.

Some information was obtained from the 10 selected features. First of all, no shape feature remained after selection. The reason might come from the fact that all of the ROIs were provided by the deep networks, which had a homogeneous shape regardless of the pCR status. Furthermore, half of the selected features were wavelet features, which might imply that some valuable information was hidden in the frequency domain. Many recent studies have also highlighted the importance of wavelet features in radiomics analysis (43–46). Finally, 8 of the 10 features were quantiles, and 1 was the standard deviation. This suggested that simply averaging the features from all of the slices per patient was insufficient compared with using multiple statistics.

To further address the thoughtful design of our study, we wanted to highlight the importance of independent training of the segmentation and radiomics model. As a model always tends to overfit more or less while training, if we use the same set to train the segmentation as well as the radiomics, the predicted ROI on the training set and validation set might have different distributions, and consequently affect the stability of the radiomics analysis. Due to the above consideration, we used a particular dataset, Seg-T, to train the segmentation model and choose the network parameters. We then applied the best model on Rad-T, In-V, and Ex-V to assure the independence between segmentation and radiomics.

Unlike other studies (47, 48) that have utilized a two-sample *t*-test as the first step to select the features, we used univariate Cox analysis. In fact, prior to the feature selection, we examined whether each feature was normally distributed by calculating the skewness and kurtosis (49). A total of 34 features did not pass this simple normality test under a significance level *α* = 0.05. Therefore, *t*-test could not be applied to all features in our study. Consequently, we utilized the concordance index as a replacement because it did not limit the distribution of data.

There were some limitations in our study. However, this was only a preliminary exploration. In the future, we will attempt further improvements. For instance, we could collect more unified standard data for analysis, and at the same time carefully choose the year of patient recruitment to avoid data mixing. In addition, we could encourage radiologists to delineate more precisely so that our segmentation network could better learn the characteristics of the suspicious regions. Additionally, our segmentation network could be adjusted and improved using techniques, such as combining different loss functions following previous works (50–53) or adding clinical characteristics for a joint analysis. In addition, if we could obtain MRI with smaller slice thickness (≤1*mm*), we could consider building a 3D model and studying the 3D radiomics features that may contain richer information of the suspicious region.

Finally, the motivation of this study was different than other related works. We wanted to explore the possibility of using a single post-nCRT T2 MRI for patients missing a pre-nCRT MRI or other modalities. In addition, we intended to improve the efficiency of the model and reduce the workload of doctors for clinical use. Our method has great potential to guide less-experienced doctors, as it does not require manual delineation of the ROI. Moreover, as our model is less restricted regarding data requirements and the prediction of the pCR was easily obtained, it can be combined with other studies for joint decision-making.

## Data Availability Statement

The raw data supporting the conclusions of this article will be made available by the authors, without undue reservation.

## Ethics Statement

The studies involving human participants were reviewed and approved by This clinical trial was approved by the Clinical Ethics Review Committee of the Sixth Affiliated Hospital of Sun Yat-sen University (2020ZSLYEC-010).

## Author Contributions

XP, FW, QZ, and YL jointly wrote the manuscript. XP, RH, and XY collected all the input sources data. XP and FW annotated the images data. FW, QZ, and YL designed the model and implemented the main algorithm and other computational analysis. XF performed a review of the manuscript on the clinical aspects. All authors contributed to the article and approved the submitted version.

## Funding

Guangdong Science and Technology Project (No. 2019B030316003) and The Sixth Affiliated Hospital of Sun Yat-Sen University Clinical Research 1010 Program [No. 1010PY (2020)-09] supported this study.

## Conflict of Interest

The authors declare that the research was conducted in the absence of any commercial or financial relationships that could be construed as a potential conflict of interest.

## Publisher’s Note

All claims expressed in this article are solely those of the authors and do not necessarily represent those of their affiliated organizations, or those of the publisher, the editors and the reviewers. Any product that may be evaluated in this article, or claim that may be made by its manufacturer, is not guaranteed or endorsed by the publisher.
